# Clinical Presentation, Diagnosis, Treatment, and Outcomes of Myocarditis in Children: A Tertiary Care Hospital Experience

**DOI:** 10.7759/cureus.57178

**Published:** 2024-03-29

**Authors:** Khadim Khan, Ijaz Hussain, Saadia Ilyas, Zaland A Yousafzai, Rida Khan, Farman Ali

**Affiliations:** 1 Pediatrics, Peshawar Institute of Cardiology, Peshawar, PAK; 2 Pediatric Cardiology, Lady Reading Hospital, Peshawar, PAK; 3 Medicine, Rehman Medical Institute, Peshawar, PAK; 4 Pediatric Cardiology, Peshawar Institute of Cardiology, Peshawar, PAK

**Keywords:** pediatric cardiology, cardiology, pediatric myocarditis, fulminant myocarditis, myocarditis

## Abstract

Background

Clinical presentation, diagnosis, and treatment of myocarditis in children can be highly challenging, and results can vary greatly. Research on the precise processes of myocardial injury, including the effects of viral infections and newly identified variables like COVID-19, is still underway. Though treatment approaches, such as immunosuppressive therapy, are still debatable, diagnostic methods such as cardiac MRI and biomarkers show promise in improving diagnostic accuracy. The purpose of this study is to describe the spectrum of pediatric acute myocarditis, assess existing therapy approaches, and develop regional guidelines based on the experience of a tertiary care institution.

Methods

Children diagnosed with acute myocarditis over a six-month period were included in this retrospective and descriptive hospital-based study. Data on demographics, clinical presentations, diagnostic tests, treatments, and results were gathered and examined. Descriptive statistics, non-parametric tests for categorical variables, and Spearman's correlation tests for continuous data were used in the statistical analysis, with a significance level of p < 0.05.

Results

Of the 99 patients included, the mean age was 2.37 years, with males making up the majority (n = 54, 54.55%). Clinical symptoms typically included shortness of breath (n = 998, 99.0%), vomiting (n = 63, 63.6%), and chest pain (n = 6, 6.1%). High levels of troponin I (n = 70, 70.7%), cardiomegaly on a chest X-ray (n = 97, 97.0%), and different degrees of ventricular dysfunction were found in the laboratory and in imaging studies. Methylprednisolone (n = 84, 84.8%) and IV immunoglobulin (n = 54, 54.5%) were the most often used treatment modalities, and there were no appreciable differences in the two treatment groups' outcomes. A weak negative association (Spearman's rho = -0.211, p = 0.036) was found in the correlation study between the administration of methylprednisolone and length of stay (LOS), indicating possible benefits in terms of shortening hospital stays.

Conclusion

This research offers a significant understanding of the clinical manifestation, treatment, and complications of acute myocarditis in children. Methylprednisolone administration seems to be linked to a shorter length of stay (LOS), despite disagreements over treatment approaches. To confirm these results and provide guidance for evidence-based management guidelines for pediatric myocarditis in our setup, more studies are necessary.

## Introduction

The Dallas criteria define myocarditis as the inflammatory cellular infiltration in the myocardium followed by cardiomyocyte necrosis of non-ischemic origin [1]. Pediatric myocarditis is a pediatric ailment most commonly linked to a prior viral infection. In children, it is the primary cause of cardiomyopathy, acquired heart failure, and heart transplants. The diagnosis is frequently overlooked until far later in the course of the illness because of the heterogeneity in presentation. Given that respiratory distress is the most common manifestation, it ought to be taken into account in the differential diagnosis of all children presenting with it [2]. The most common viruses found in endomyocardial biopsies at the moment are adenoviruses and parvovirus B19. The etiology involves both an immunological response against cardiac epitopes and direct viral myocardial damage [3]. Following its initial report in December 2019 from Wuhan, China, the coronavirus disease 2019 (COVID-19) has become a major global source of mortality and morbidity. Numerous case reports have connected COVID-19 to myocarditis; however, the precise mechanism of cardiac damage is still being studied [4]. There is great variation in the clinical presentation; it might be asymptomatic or result in unexpected death. The key is a high index of suspicion. Research supporting the use of cardiac MRI and serum N-terminal B-type natriuretic peptide levels as adjuvants to clinical diagnosis is beginning to emerge. These could eventually lessen the need for invasive techniques, including endomyocardial biopsy, which is still the gold standard [5]. A virus genome or persistent intramyocardial inflammation can cause 20% of cases of acute myocarditis to develop into dilated cardiomyopathy. The prognosis is dire for both acute myocarditis (10-year survival rate: 45%) and dilated cardiomyopathy (the primary cause of heart transplantation). The application of immunohistochemical and molecular-biological diagnostic techniques to endomyocardial biopsy material allowed for the first time the etiopathogenic differentiation of dilated cardiomyopathy [6]. The WHO and the International Society and Federation of Cardiology (ISFC) define dilated cardiomyopathy as a myocardial disease characterized by severe enlargement of the left or right ventricle, which results in a decrease in the heart's systolic function (decrease of contractility) and later congestive heart failure. A complex diagnostic flow diagram must be followed in order to differentiate between primary cardiomyopathies, which are defined as intrinsic cardiac muscle diseases, and cardiomyopathies, which are the result of chronic inflammatory myocarditis (chronic persistent myocarditis, chronic immune myocarditis, and chronic viral heart disease). The bioptic specimens must be analyzed using a light microscope (according to Dallas criteria), an electron microscope, and afterward immunobiologically and immunohistochemically (in situ hybridization) [7]. 

Although remarkable advances in diagnosis, understanding of pathophysiological mechanisms, and treatment of acute myocarditis were gained during the last years, no standard treatment strategies could be defined as yet, apart from standard heart failure therapy and physical rest. In severe cases, mechanical support or heart transplantation may become necessary [8]. 

A retrospective study was conducted in China to investigate the clinical efficacy of coenzyme Q10 (CoQ10) plus trimetazidine (TMZ) in treating acute viral myocarditis (AVMC) and the combination's influence on the oxidative stress markers and patients' quality of life (QoL). CoQ10 plus TMZ yielded favorable clinical effectiveness in the treatment of AVMC, and it effectively promoted cardiac function recovery, alleviated oxidative stress and inflammatory reactions, and bolstered patients' QoL [9]. 

Immunosuppressive therapy such as corticosteroids has been used in patients. In experimental models and some uncontrolled cases of myocarditis, IV immunoglobulin (IVIG) has been shown to have antiviral and immunomodulatory effects. However, the efficacy of corticosteroids and IVIG remains controversial [10]. The best practice for treating myocarditis is also controversial. IVIG and steroids are both anti-inflammatory treatments commonly used though data supporting their benefits is limited and largely comes from adult studies [11]. 

The aim of our study is to define the spectrum of acute myocarditis in children and its outcome with current management.

## Materials and methods

Study

The study was conducted at the Peshawar Institute of Cardiology, Peshawar, Pakistan. All pediatric patients admitted with a diagnosis of acute myocarditis over the six months prior were included in this retrospective and descriptive hospital-based study. Before the start of the study, the Institutional Review Board (IRB) of the Peshawar Institute Of Cardiology granted ethical permission for this investigation with approval reference number 2884. Informed consent was also acquired. This study’s data collection was done over a period of six months from July 2023 to December 2023. 

Participants

Pediatric patients diagnosed with acute myocarditis who were younger than eighteen years old and admitted, both male and female, were included in the study. On the other hand, children who were admitted with congenital cardiac disease for any procedure or treatment and those who had already been diagnosed with dilated cardiomyopathy (DCM) were excluded from the study. The sample size included all eligible patients who met the inclusion criteria throughout the stated study period.

Data collection

Demographic information, such as age and gender, together with clinical presentation, diagnostic investigations, treatment methods, and outcomes, were gathered by reviewing patient records. All data were anonymized and managed with absolute confidentiality in compliance with institutional rules. 

Data analysis

Statistical analysis was performed using the IBM SPSS Statistics for Windows, Version 26, (Released 2019; IBM Corp., Armonk, New York, United States). Descriptive statistics were employed to summarize demographic and clinical characteristics. Categorical variables were expressed as frequencies and percentages, whereas continuous variables were reported as means with standard deviations. Before running correlation tests, the test of normality was performed on continuous variables like age, length of stay (LOS), and weight to check for the distribution. Kolmogorov-Smirnova and Shapiro-Wilk tests of normality were significant (p < 0.05), which indicates that these variables were not normally distributed within the sample population. Hence non-parametric tests, such as Spearman’s correlation test, were used for continuous data, and the Chi-square test was used for the categorical variables. A p-value less than 0.05 was deemed to be statistically significant.

## Results

A total of 99 patients were included in the study. Out of the 99 patients in this study, 54 (54.55%) were male, and 45 (45.45%) were female, revealing a slightly higher percentage of males. The mean age was 2.37 years, with a standard deviation of 1.95 years. The mean weight of the group of patients was found to be 12.05 kg, with a standard deviation of approximately 6.31 kg. The study's findings revealed that the average length of stay for patients admitted with myocarditis was 9.59 days with a standard deviation of 16.87 days. Of the 99 patients in the study, 96 patients (97.0%) were discharged home after receiving therapy, and three patients (3.0%) expired during hospital stay. According to clinical symptoms amongst the patients, 99.0% (n = 98) of individuals reported having shortness of breath; it was the most often reported symptom. Vomiting was also common with 63.6% (n = 63) reporting experiencing it. Only 6.1% (n = 6) of the group reported having chest pain, compared to 32.3% (n = 32) of those who reported having abdominal pain. 

On clinical examination, 94.9% (n = 94) of individuals had respiratory distress. In 38.4% (n = 38) of participants, signs of congestive heart failure were identified, and in 6.1% (n = 6) of cases, signs of shock were present. Hepatomegaly was seen in 97.0% (n = 96) of instances, while gallop rhythm was appreciated in 98.0% (n = 97) of individuals. Of the individuals, 96.0% (n = 95) had a weak pulse. Surprisingly, there were no arrhythmias among the individuals. 

The troponin I levels were raised in 70.7% (n = 70) of the patients; in the laboratory examinations, out of the total number of patients, 20 (20.2%) had levels between 100-200, 24 (24.2%) had levels between 33-100, 26 (26.3%) had levels above 200, and 29 (29.3%) had levels below 33. Of the patients with chest X-ray results, 97 (97.0%) showed cardiomegaly, and only three (3.0%) had normal results. An examination of the ejection fraction showed that eight patients (8.1%) fell between 45 and 60, 29 patients (29.3%) fell below 30, and 62 patients (62.6%) fell between 30 and 45. Additionally, six patients (6.1%) of the cases had intracardiac thrombus, while 93 patients (93.9%) did not exhibit thrombus. Moreover, only three individuals (3.0%) had effusion found in them. Of the patients, one (1.0%) had mild dysfunction, 16 (16.2%) had moderate dysfunction, and 82 (82.8%) had severe dysfunction. These findings combined give useful insights into the cardiac profile of the research group, underlining the prevalence of cardiac problems and the demand for focused therapies and management measures. 

Regarding management and drug administration, 54.5% (n = 54) of patients received IVIG, while 84.8% (n = 84) of patients received methylprednisolone. Milrinone was administered to only 5.1% (n = 5) of the patients, while dobutamine and dopamine were given to the majority of patients 92.9% (n = 92) and 65.7% (n = 65), respectively. Only 5.1% (n = 5) of patients received epinephrine, which was rarely used. Table 1 gives a comprehensive summary of demographic characteristics, clinical presentation, examination findings, laboratory results, radiological findings, echocardiographic assessment, and management strategies in patients diagnosed with acute myocarditis.

**Table 1:** Summary of clinical presentation, examination findings, laboratory results, radiological findings, echocardiographic assessment, and management strategies in pediatric patients with acute myocarditis. The data has been represented as count/frequency (N), and percent (%).

Variables		Frequency	Percent
Gender	Male	54	54.55%
	Female	45	45.45%
Symptoms			
Shortness of breath	Not Present	1	1.0%
	Present	98	99.0%
Vomiting	Not Present	36	36.4%
	Present	63	63.6%
Abdominal pain	Present	32	32.3%
	Not Present	67	67.7%
Chest pain	Present	6	6.1%
	Not Present	93	93.9%
Clinical signs			
Respiratory distress	Not Present	5	5.1%
	Present	94	94.9%
Congestive cardiac failure	Present	38	38.4%
	Not Present	61	61.6%
Shock	Present	6	6.1%
	Not Present	93	93.9%
Gallop	Not Present	2	2.0%
	Present	97	98.0%
Hepatomegaly	Not Present	3	3.0%
	Present	96	97.0%
Weak pulse	Not Present	4	4.0%
	Present	95	96.0%
Arrhythmias	Present	0	0.0%
	Not Present	99	100.0%
Investigations			
Troponin I in ng/L	100-200	20	20.2%
	33-100	24	24.2%
	>200	26	26.3%
	Less than 33	29	29.3%
Chest X-ray	Normal	3	3.0%
	Cardiomegaly	96	97.0%
Ejection fraction in percentage	45-60	8	8.1%
	<30	29	29.3%
	30-45	62	62.6%
Intracardiac thrombus	yes	6	6.1%
	no	93	93.9%
Effusion	yes	3	3.0%
	no	96	97.0%
Dysfunction	Mild	1	1.0%
	Moderate	16	16.2%
	Severe	82	82.8%
Treatment			
Dobutamine	Not Received	7	7.1%
	Received	92	92.9%
Dopamine	Not Received	34	34.3%
	Received	65	65.7%
Milrinone	Received	5	5.1%
	Not Received	94	94.9%
Epinephrine	Received	5	5.1%
	Not Received	94	94.9%
IV immunoglobulin	no	45	45.5%
	Received	54	54.5%
Methylprednisolone	Not Received	15	15.2%
	Received	84	84.8%
Outcome			
Outcome	Discharged	96	97.0%
	Expired	3	3.0%

Between patients receiving IV methylprednisolone and IVIGs, there were no statistically significant differences in death rates, or need for dual support (defined as the administration of two or more agents, such as dobutamine and dopamine) observed in our study. Through statistical analyses utilizing the chi-square test (p-value > 0.05) for mortality rates and the need for dual assistance, this conclusion was achieved. 

Methylprednisolone administration was found to be associated with LOS, as evidenced by a -0.211 (p = 0.036) Spearman's rho correlation value showing a weak negative correlation which means that patients who received methylprednisolone may have tended to have shorter LOS. In comparison, there was a smaller and non-statistically significant negative correlation between IVIG administration and LOS (Spearman's rho = -0.130, p = 0.199) (Table 2).

**Table 2:** Correlation is significant at the 0.05 level (two-tailed). Correlation coefficients and p-values for both methylprednisolone and IV immunoglobulin with LOS indicate the strength and significance of their respective relationships. A p-value less than 0.05 was deemed significant.

Variables		Length of stay (LOS)			
		Mean	Standard deviation (SD)	Correlation coefficient (Spearman's rho)	p-value
Methylprednisolone	Yes	8	5	-0.211*	0.036
	No	6	4		
IV Immunoglobulin	Yes	8	5	-0.130	0.199
	No	7	5		

## Discussion

Myocarditis, a pediatric illness often caused by viral infections, is characterized by inflammatory cellular infiltration in the myocardium and subsequent cardiomyocyte destruction. This condition can result in acquired heart failure, cardiomyopathy, and cardiac transplants. Various manifestations often cause delays in diagnosis, with respiratory distress being the most common [1,2]. Endomyocardial biopsies frequently reveal the presence of parvovirus B19 and adenoviruses, with inflammatory responses and direct viral myocardial injury playing a role in the pathophysiology [3]. Although case reports have connected COVID-19 to myocarditis, the precise mechanism of cardiac damage is still being studied [4]. 

A strong index of suspicion is necessary since the clinical spectrum of myocarditis varies greatly, ranging from asymptomatic to abrupt death. New diagnostic technologies like cardiac MRI and serum N-terminal B-type natriuretic peptide levels may lessen the need for invasive techniques like endomyocardial biopsy [5]. Twenty percent of cases of acute myocarditis might proceed to dilated cardiomyopathy, which has a poor prognosis and occasionally calls for heart transplantation. Complex diagnostic methods are required to differentiate between primary cardiomyopathies and those developing from chronic inflammatory myocarditis [6,7]. In extreme cases, mechanical assistance or transplantation may be necessary; however, standard treatment techniques for acute myocarditis remain unclear despite recent advancements [8]. 

Our study revealed a significantly lower average age of 2.37 years and practically equal distribution of male and female patients compared to the study carried out in Pakistan in 2022 [12], which revealed an average age of 6.8 ± 2.1 years and a male-to-female ratio of 67.6% to 32.4%. Comparing our results to the 2016 Indian study, which found 43 individuals with a possible diagnosis of myocarditis, we also found differences in the mean age and gender distribution. The Indian study indicated a mean age of 6.1 ± 3.4 years and a male-to-female ratio of 1.2:1 [13] in contrast to our study's lower mean age and a closer approximation of gender distribution. Variations in illness prevalence among regions, genetic predispositions, environmental factors, diagnostic standards, and healthcare accessibility are some of the causes that could be causing these variances. Furthermore, variations in research populations, approaches, and sample sizes could also explain the discrepancies noted. 

In our study, the most common symptom was shortness of breath (99%) while chest pain (6.1%) was the least common symptom; a study in India reported similar results with shortness of breath as being the most common symptom reported by 83.7% of the patients, while 39.5% of the patients reported chest pain [13]. A study by Durrani et.al. also reported shortness of breath as the most common symptom [14]. This underscores the significance of identifying dyspnea in pediatric patients as a potential marker of myocarditis. On the other hand, the comparatively lower prevalence of chest pain highlights the unpredictability of symptom presentation and the necessity for doctors to keep a high index of suspicion, particularly in the absence of traditional symptoms like chest pain.

In our study group, respiratory distress was the most common clinical symptom present in 94.9% of the patients; this is consistent with findings from earlier research studies [14,15]. Early presentation and diagnosis are critical to improving the prognosis and lowering the risk of adverse events during the hospitalization of children with myocarditis. It also emphasizes the need to rapidly recognize essential symptoms. None of the patients in our group developed arrhythmias; this is in contrast to other studies. In one instance, a study on the spectrum of arrhythmias and outcomes in pediatric myocarditis discovered that a considerable proportion of these patients had aberrant ECG readings, including sinus tachycardia [16]. Different arrhythmia patterns have also been noted in viral myocarditis patients, with ventricular arrhythmias aggravating the disease's clinical course and affecting prognosis [17]. Disparities in arrhythmia presentation among patient cohorts may potentially be attributed to the time of presentation, variations in viral strain, and host immunological response. Further research into the temporal progression of myocarditis and its association with arrhythmia development is needed to clarify these potential mechanisms. 

In our group of patients with myocarditis, cardiomegaly on X-rays was identified in 97% of the patients, The proportion of individuals exhibiting cardiomegaly on chest X-rays in pediatric myocarditis differs between research studies. Studies show that between 52.9% and 85.7% of pediatric myocarditis patients have cardiomegaly on their chest X-ray. [18,19]. According to Durani et al., cardiomegaly was present in 63% of the patients with viral myocarditis [14]. The significance of using chest X-rays as a diagnostic tool in recognizing heart enlargement associated with this ailment is highlighted by the high prevalence of cardiomegaly on X-rays in pediatric myocarditis patients. 

When myocarditis is clinically suspected, echocardiography is still the most helpful diagnostic procedure [19]. According to echocardiography findings, a large number of patients had LV dysfunction, 82.8% had severe dysfunction, while 16.2% had moderate dysfunction. Most of the patients (91.9%) had an ejection fraction of less than 45 percent. Similarly, atypical echocardiograms were discovered in 60 out of 61 (97%) individuals, according to Durani et al. [14]. Furthermore, a retrospective study conducted in 2019 revealed that up to 50% of patients with pediatric myocarditis had left ventricular (LV) systolic dysfunction, making it a common characteristic of the condition. About 14% of the individuals demonstrated significant impairment. Furthermore, a strong predictor of poor outcomes in pediatric myocarditis is an LV ejection fraction (LVEF) of less than 30% on echocardiography at the time of admission [20]. Atypical echocardiographic abnormalities were common, which is in line with earlier research and highlights the value of echocardiography in the diagnosis of myocarditis. 

Patients with acute myocarditis may have high levels of troponin I and T, which are widely used in pediatric settings to detect troponin leakage. In myocarditis confirmed by biopsy, troponin does not seem to be a sensitive or specific enough marker. It is often found at extremely high concentrations when it is raised [21]. This is in accordance with our study, in which 70.7% of the patients had raised troponin I levels. 

In hospitalized patients, we observed a relationship between the LOS and pharmacological interventions, particularly methylprednisolone and IVIG. Methylprednisolone administration and the LOS showed a statistically significant weak negative correlation (p = 0.036) in our analysis, suggesting that patients on methylprednisolone tended to have shorter LOS. 

In contrast, there was a weaker and non-statistically significant (p = 0.199) correlation between IVIG treatment and the LOS. These results point to a possible link between the LOS and methylprednisolone, which suggests that administering it could shorten hospital stays. Nevertheless, since the LOS may be influenced by other factors, care must be taken when interpreting these findings as significant. Additional investigation is necessary to have a comprehensive understanding of the clinical effects of pharmaceutical therapies on hospital outcomes and to establish a causal relationship. This can be achieved through the implementation of prospective studies and randomized controlled trials. This observation aligns with the results of Yao and Zhan's 2023 meta-analysis, which encompassed six trials comprising 604 pediatric patients diagnosed with acute myocarditis. When compared to anti-failure medication, the results of the meta-analysis showed that corticosteroid therapy did not significantly lower the risk of mortality from acute myocarditis. On the other hand, patients treated with corticosteroids showed a significant improvement in LV function, as shown by an increase in LVEF. Despite these encouraging findings, it's crucial to acknowledge the significant variability seen in the meta-analyses about the likelihood of achieving clinical objectives like cardiac transplantation or mortality, as well as the absence of changes in LV end-diastolic diameter (LVEDD) [22]. 

The fact that patients treated with methylprednisolone in our trial had shorter hospital stays than those treated with IVIG points to the possible advantages of its immunomodulatory and anti-inflammatory properties. The effects of steroids on cardiac tissue may hasten the improvement of clinical outcomes. However, other factors that affect results include adverse events, treatment timing, and individual reactions. 

There is disagreement over the use of intravenous immunoglobulin (IVIG) in children with myocarditis due to inconsistent data. Some research shows benefits, but others don't. A 2019 meta-analysis by Yen et al suggested that IVIG may not improve pediatric myocarditis. In this 13-study analysis, the IVIG group initially had a higher survival rate than the non-IVIG group, but further adjustments made the difference insignificant. Age and gender did not significantly affect the survival rate in the meta-regression, underlining IVIG's complexity [23]. In contrast, IVIG could be used to treat pediatric myocarditis. According to a meta-analysis that was published on the National Center for Biotechnology Information (NCBI) portal in 2019, IVIG may help infants with myocarditis survive longer and have a higher LVEF. According to this study, children receiving IVIG had lower death or heart transplantation rates than their non-treated counterparts [24].

These results highlight the need for additional research, such as prospective randomized controlled trials, to clarify the best course of action for treating pediatric myocarditis and improve clinical management techniques. 

Limitations

It is important to recognize that there are a number of limitations even if our study offers insightful information on treating pediatric myocarditis. First of all, the retrospective nature of our study design introduced biases and restrictions to the data gathering and analysis process. Subsequently, the single-center design and rather limited sample size might restrict how far our results can be applied. Moreover, our capacity to evaluate the sustainability and duration of treatment effects was hampered by the absence of long-term follow-up data. Furthermore, our findings may not be applicable in other clinical situations due to differences in treatment methods and patient characteristics among other healthcare facilities. Lastly, conclusive findings of treatment efficacy and safety were precluded by the lack of a direct comparison between various treatment modalities in our study.

## Conclusions

Our findings highlight the urgent need for additional studies to improve the way pediatric myocarditis is managed. The observed variations in the course of treatment and the absence of agreement on the best course of action for therapy serve to emphasize how complicated this illness is. The comparative effectiveness, safety profiles, and long-term results associated with various treatment approaches must be clarified through prospective randomized controlled trials. Future research efforts could potentially close these information gaps, improve patient outcomes, and lessen the burden that pediatric myocarditis imposes on both the patients and the healthcare system. 
